# Chitosan functionalized nanocochleates for enhanced oral absorption of cyclosporine A

**DOI:** 10.1038/srep41322

**Published:** 2017-01-23

**Authors:** Min Liu, Xiaoming Zhong, Zhiwen Yang

**Affiliations:** 1 Department of Pharmacy, Songjiang Hospital Affiliated Shanghai First People’s Hospital, Shanghai Jiao Tong University, Shanghai, China; 2 Urology Department, First Affiliated Hospital of Gannan Medical University, Gannan Medical University, Ganzhou, China; 3 Jiangxi Province Tumor Hospital, Nanchang, China

## Abstract

It remains a significant challenge to overcome the poor permeability of cyclosporine A and enhance its oral absorption. In this study, we have identified a positively charged chitosan that is able to induce coiling up of anionic lipids to form nanocochleates with an average size of 114.2 ± 0.8 nm, without the need for calcium ions. These functional chitosan-induced nanocochleates enhanced gastrointestinal absorption of cyclosporine A, up to a 3-fold increase in oral bioavailability. A fluorescence-labeling study confirmed that absorption mainly occurred in the duodenum and jejunum. Transport studies indicated that uptake of chitosan-induced nanocochleates by Caco-2 cells was by clathrin- and caveolae-mediated endocytosis, but not by macropinocytosis. Furthermore, three cellular tight junction proteins, ZO-1, F-actin and claudin-4, were significantly down-regulated, suggesting that chitobiose-induced nanocochleates are able to reconstruct and open tight junctions in intestinal epithelial cells to enhance drug absorption. In summary, these novel bifunctional chitosan-induced nanocochleates appear to have potential to facilitate oral delivery of cyclosporine A.

Cyclosporine A (CsA) is a powerful immunosuppressive agent that has been widely used to attenuate or prevent allograft rejection after various organ transplantations in clinical practice. Due to its poor solubility and permeability, it is difficult to produce an oral formulation of CsA. Two oral self-microemulsion formulations of CsA, marketed as Sandimmune^®^ and Neoral^®^, composed of a high concentration of polyoxyethylated castor oil (CremophorEL^®^, up to 38 w/w%), are currently available. However, CremophorEL^®^ has been identified as an unsafe oil composition that exerts some adverse effects *in vivo*, such as hypersensitivity, gastrointestinal, anaphylactoid and nephrotoxic reactions. Extensive effort has therefore been devoted to the design and development of an effective and safe oral formulation of CsA.

Oral formulations provide many advantages, including improved patient compliance, lower medical expenses and fewer side effects^1^. However, there are many drugs that have not been used orally in clinical practice, likely due to their poor membrane permeability and low aqueous solubility, which limits transport from the intestinal tract to the blood circulation^2^. Advances in the understanding of the gastrointestinal tract have led to some effective lipid-based carriers to enhance drug solubility and gastrointestinal permeability^3^,^4^.

Nanocochleates are stable phospholipid-cation precipitates with multilayered cylindrical structures, composed of positively charged calcium ions and negatively charged phospholipid^5^. When drugs are incorporated into nanocochleates, the unique multilayered cylindrical structures provide protection from drug degradation in hazardous environmental conditions, resulting in enhanced oral bioavailability^6^,^7^. To our knowledge, it appears impossible to develop an oral formulation of amphotericin B (AmB) because of its low solubility and permeability. Interestingly, an oral AmB-loaded nanocochleate formulation was in development by Biodelivery Sciences, Inc. (Raleigh, NC, USA), who released Phase I trial data in February 2009 showing a positive therapeutic effect equivalent to intravenous therapy^8^. This result suggested that nanocochleates could be a promising lipid-based delivery system for oral administration.

Chitosan is an attractive material for oral delivery that exhibits permeation enhancement^9^, mucoadhesion^10^, and P-gp inhibition^11^. In this study, we attempted to develop a novel chitosan-induced nanocochleate system to assemble nanocochleate structures without the requirement for calcium ions. Unlike calcium ion-induced nanocochleates reported previously, chitosan-induced nanocochleates provide the unique property of enhanced intestinal absorption of drugs because of the presence of chitosan (Fig. 1). Herein, we have evaluated the effects of chitosan-induced nanocochleates on the *in vivo* and *in vitro* transport efficiencies of CsA. Cellular transport mechanisms across the intestinal barrier were also investigated in cellular and SD rat models.

## Materials and Methods

### Materials

CsA was obtained from Yuanye Bio-Technology Co., Ltd (Shanghai, China). Dioleoylphosphatidyl serine (DOPS, >90%) was obtained from Lipoid GmbH (Ludwigshafen, Germany). Chitobiose was purchased from Sigma-Aldrich (Darmstadt, Germany). ZO-1, F-actin, andclaudin-4 antibodies were purchased from Abcam (Cambridge, MA, USA).

### Preparation of nanocochleates

Calcium ion-induced nanocochleates containing CsA were prepared as previously described^12^,^13^. CsA in ethanol (0.5 mg/mL) was added to DOPS in 100% chloroform at a molar ratio of 10:1. The organic solvent was then evaporated to dryness under reduced pressure using a rotary evaporator to form a thin lipid film. The film was rehydrated with PBS buffer (pH7.4) at a concentration of 5 mg of lipid/mL, and the obtained liposome suspension was sonicated for 10 min with a probe sonicator. Nanocochleates were formed by slow addition of calcium chloride (0.1 mM) to the CsA-loaded liposome suspension with stirring at 1000 rpm for 5 min. Nanocochleate formulations were extruded through a 0.45 μm Millex™-GP filter unit (Millipore Corporation, Bedford, MA, USA) to remove larger nanocochleates.

Chitobiose-induced nanocochleates containing CsA were prepared similarly. Briefly, nanocochleates were formed by slow addition of chitobiose solution (2 μg/mL) to the CsA-loaded liposome suspension.

Coumarin-6-labeled nanocochleates were prepared similarly. Briefly, coumarin-6-loaded liposomes were used to form nanocochleates by slow addition of calcium chloride.

### Characterization of nanocochleates

#### Transmission electron microscopy

The morphology of CsA-loaded nanocochleates was observed using transmission electron microscopy (JEM-1400, JEOL Ltd., Tokyo, Japan) at 200 kV after phosphor-tungstic acid negative staining. The nanocochleate suspension was diluted with phosphate buffer, adsorbed onto a 300-mesh holey copper grid, and dried under ambient conditions. After blotting with 1% phosphor-tungstic acid (w/v, pH 6.5), samples were ready for TEM observation.

#### Particle size measurements

The mean z-average diameter and polydispersity index (PDI) were established by photon correlation spectroscopy using a Zetasizer Nano ZS90 (Malvern Instruments, Malvern, UK) at 25 °C. CsA-loaded nanocochleates were diluted in double-distilled water. All samples were analyzed in triplicate.

#### Encapsulation efficiency

The encapsulation efficiency of CsA-loaded nanocochleates was determined using a Sephadex microcolumn separation method. CsA-loaded nanocochleate suspension (1 mL) was applied to a Sephadex G-50 gel microcolumn to separate free drug. The filtered dispersion was then diluted with methanol to disrupt the nanocochleates. The CsA concentration was determined using high performance liquid chromatography (HPLC).

#### *In vitro* drug release

The *in vitro* release behavior of CsA-loaded nanocochleates was evaluated using a membrane dialysis method. Samples equivalent to 5 mg of CsA were dispersed and sealed in a dialysis bag (MWCO 10,000 Da). Dialysis bags were then immersed in 900 mL of release medium (pH 7.4, PBS buffer), maintaining a revolution speed of 100 rpm at 37 °C. At time intervals of 0.5, 1, 2, 3, 4, 5, and 6 h, 1.5 mL samples of the release medium were collected and replaced with fresh medium. Drug content in the release medium was determined using HPLC.

#### *In vivo* pharmacokinetics study

Male Sprague-Dawley rats weighing 180–200 g were housed under standard temperature and humidity. The rats were randomly divided into three groups (N = 6 for each group).

1. Group I was treated orally with CsA raw drug, equivalent to 50 mg/kg CsA.
2. Group II was treated orally with CsA-loaded nanocochleates induced by calcium ions, equivalent to 50 mg/kg CsA.
3. Group III was treated orally with CsA-loaded nanocochleates induced by chitobiose, equivalent to 50 mg/kg CsA.

After administration of a single oral dose, blood samples at 0.5, 1, 1.5, 2, 3, 4, 6, 8, 12 and 24 h were collected into heparinized micro-centrifuge tubes. Then, 400 μL of diethylether-methanol (95:5) solution was added to 200 μL of plasma to remove plasma proteins. Subsequently, 20 μL samples of the supernatants were injected into a Phenomenex Luna C-18 column at 55 °C to determine drug content. CsA was eluted at a flow rate of 1 mL/min using a mobile phase consisting of methanol and purified water (90:10, v/v).

#### Distribution in the intestinal tract

Calcium ion-induced and chitobiose-induced nanocochleates were fluorescently labeled with coumarin-6 as described in the section on nanocochleate preparation. Briefly, coumarin-6-labeled nanocochleates and coumarin-6 solution (negative control) were administered to SD rats by oral gavage. At 2 h after oral administration, the rats were sacrificed to collect duodenum, jejunum, and ileum segments. Samples were fixed on cationic resinous slides with 4% formalin at room temperature. Finally, the samples were labeled with 4,6-diamidino-2-phenylindole dihydrochloride (DAPI, nuclei dye) for 5 min and visualized under a confocal laserscanning microscope.

### Transport mechanism across Caco-2 cells

#### Transport across Caco-2 cells

CsA-loaded nanocochleates were added to the apical side. At predetermined time intervals, 0.2 mL solution was taken from the basolateral side and replaced with fresh medium. The concentration of CsA was analyzed by HPLC.

#### Cholesterol depletion

Methyl-β-cyclodextrin (10 mmol/L) was added into Caco-2 cells and pre-incubated for 1 h at 37 °C. A transport experiment of CsA-loaded nanocochleates was then performed in the presence of 1 μg/mL lovastatin for 2 h at 37 °C.

#### Inhibition of caveolae-mediated endocytosis

Filipin (10 μg/mL) was added into Caco-2 cells and pre-incubated for 1 h at 37 °C. A transport experiment of CsA-loaded nanocochleates was then performed in the presence of 10 μg/mL of filipin for 2 h at 37 °C.

#### Inhibition of clathrin-mediated endocytosis

Chlorpromazine (10 μg/mL) was added into Caco-2 cells and pre-incubated for 1 h at 37 °C. A transport experiment of CsA-loaded nanocochleates was then performed in the presence of 10 μg/mL of chlorpromazine for 2 h at 37 °C.

#### Inhibition of macropinocytosis

Amiloride (550 mM) was added into Caco-2 cells and pre-incubated for 1 h at 37 °C. A transport experiment of CsA-loaded nanocochleates was then performed in the presence of 550 mM amiloride for 2 h at 37 °C.

#### Western Blot

Chitobiose-induced nanocochleates and PBS buffer solution were respectively added into Caco-2 cells and incubated for 2 h at 37 °C. Western blotting was used to investigate the expression of three cell tight junction proteins in Caco-2 cells. In brief, samples were incubated with polyclonal antibodies to ZO-1, F-actin, and claudin-4 overnight at 4 °C, and then with secondary antibody for 60 min at 37 °C. Finally, western blot bands were captured using a Gel imaging system (BioRad, Hercules, CA, USA).

#### Cell Proliferation Assay

Blank nanocochleates at concentrations of 10, 50, 100, and 200 μg/mL were respectively added into Caco-2 cells. The number of viable cells in the plate was determined at 0, 12, 24, and 48 h using cell counting Kit-8 (CCK-8) reagent.

#### Statement

All animal care and experimental procedures were approved by the committee for Ethical Issues in the First People’s Hospital, Shanghai Jiaotong University. All methods were conducted in accordance with the Guidelines for Experiments of Shanghai Jiaotong University.

## Results

### Chitobiose-induced nanocochleates

Preparation of nanocochleates without calcium ions was optimized by screening chitosan derivatives. Five water-soluble chitosan derivatives (chitobiose, chitotetraose, low molecular weight chitosan, carboxymethyl chitosan, and chitosan oligosaccharide lactate) were used to produce nanocochleates. Calcium ion-induced nanocochleates are formed within minutes simply by mixing in the presence of negatively charged lipid, characterized as precipitate and aggregate. In this study, only chitobiose led to similar assembly behavior without a requirement for divalent cations. Thus, nanocochleates were formed by the slow addition of chitobiose, but not chitotetraose, low molecular weight chitosan, carboxymethyl chitosan, or chitosan oligosaccharide lactate.

### Transmission Electron Microscopy

**Figure 2:**
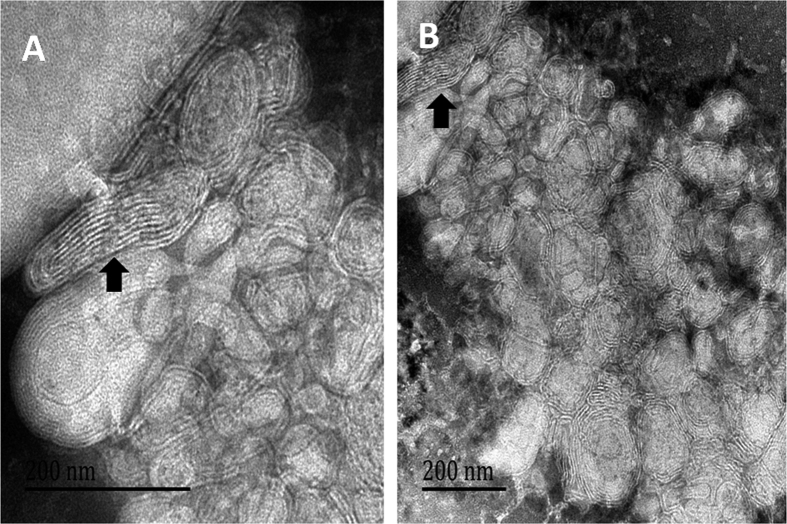
TEM image of nanocochleates. (**A**) calcium ion-induced nanocochleates; (**B**) chitobiose-induced nanocochleates.

The morphology and structure of chitobiose-induced nanocochleates were validated by transmission electron microscopy (TEM). Figure 2A shows that calcium ion-induced nanocochleates were characterized by multilayered cylindrical structures, rolled-up structures and elongated tubular structures. Figure 2B shows chitobiose-induced nanocochleates with a typical multilayered cylindrical structure similar to that of calcium ion-induced nanocochleates, indicative of the assembly of unique multilayered cylindrical structures without the requirement for divalent cations.

### CsA incorporated in nanocochleates

The CsA encapsulation efficiency of chitobiose-induced nanocochleates was evaluated by adding various amounts of CsA at lipid/drug ratios of 50:1, 10:1 and 5:1(w/w). As seen in Table 1, encapsulation efficiency was 71.8% ± 3.4%, 81.4% ± 2.0%, and 60.8% ± 0.4%, respectively. A lipid to drug ratio of 10:1 gave the highest encapsulation efficiency, so was subjected to the following studies.

### Particle Size Distribution

This study investigated the influence of the amount of cationic crosslinking agent on the nanocochleate size distribution (Table 2). CsA-loaded nanocochleates were prepared by adding different quantities of calcium chloride (200, 400, 600 μL) into 10 mL of liposomal suspension. The mean diameter of different nanocochleate dispersions was in the range of 104.0–113.5 nm with reproducible particle size and size distribution. Further increasing the quantity of calcium chloride led to nanocochleates in the nanometer to micrometer ranges, along with a tendency toward aggregation and sedimentation. The influence of chitobiose on the particle size of chitobiose-induced nanocochleates containing CsA was also investigated, and similar results to those for calcium ion-induced nanocochleates were observed. Based on these results, it can be recommended that chitobiose-induced nanocochleates containing CsA with nanometer-sized particles can be produced by the slow addition of 1000 μLchitobiose.

### *In vitro* release

**Figure 3:**
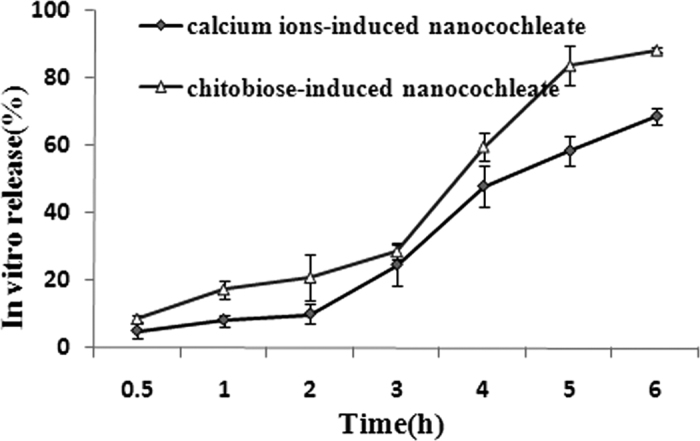
*In vitro* release profiles of CsA-loaded nanocochleates in PBS buffer at pH7.4 (N = 6).

Figure 3 shows the release of CsA from chitobiose- and calcium ion-induced nanocochleates. After 6 h, approximately 88.5% of the CsA was released from chitobiose-induced nanocochleates, while only around 68.9% of the entrapped CsA was released from calcium ion-induced nanocochleates. The release rate for chitobiose-induced nanocochleates was significantly higher than that for calcium ion-induced nanocochleates (*P* < 0.05).

#### Pharmacokinetic studies

**Figure 4:**
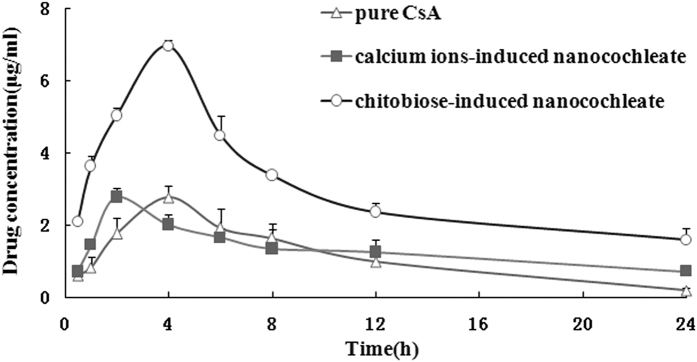
Mean plasma concentrations after a single oral dose to rats (N = 6).

The oral bioavailability of CsA formulated in chitobiose-induced nanocochleates was investigated in SD rats and compared to that of a calcium ion-induced nanocochleate formulation and pure CsA. Figure 4 shows the plasma concentration profiles of CsA following single-doseoral administration of the three formulations to rats. Plasma CsA levels for the two nanocochleate formulations were sustained for 24 h, while the drug level after dosing pure CsA was below the detection limit at 24 h. The relative oral bioavailability of CsA formulated in calcium ion-induced nanocochleates was almost equivalent to that of pure CsA. It is worth noting that the chitobiose-induced nanocochleate formulation was found to generate a higher initial plasma concentration compared to that of the calcium ion-induced nanocochleate formulation. The relative oral bioavailability with the chitobiose-induced nanocochleate formulation was about 3-foldhigher (*P* < 0.05) than that of the calcium ion-induced nanocochleate formulation.

### Biodistribution of chitobiose-induced nanocochleates

**Figure 5:**
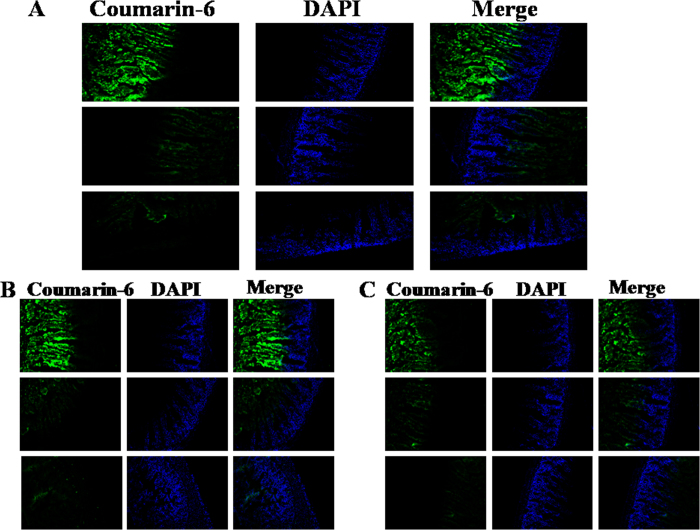
Fluorescence micrographs of rat intestine. Line 1, chitobiose-induced nanocochleates containing coumarin-6; Line 2, calcium ion-induced nanocochleates containing coumarin-6; Line 3, free coumarin-6 solution. (**A**) duodenum; (**B**) jejunum; (**C**) ileum. DAPI (blue) and coumarin-6 (green) were observed.

Compared to the calcium ion-induced nanocochleates containing coumarin-6 and free coumarin-6 solution groups, increased fluorescence of chitobiose-induced nanocochleates containing coumarin-6 was observed in different rat intestinal segments. The highest absorption was observed in the duodenum and jejunum, and lower absorption in the ileum (Fig. 5). The distribution of chitobiose-induced nanocochleates in the duodenum segment was 1.8- and 3.9-fold higher than for calcium ion-induced nanocochleates and free coumarin-6 solution, respectively. In addition, strong fluorescence of chitobiose-induced nanocochleate was found not only at the intestinal mucosal surface, but many green fluorescence signals were visible deep within the small intestine villi. As a result, chitobiose-induced nanocochleates could rapidly adhere to the intestinal mucosal layer and then pass through the intestinal tract into the systemic circulation, effectively improving the absorption of insoluble CsA.

### Transport mechanism across Caco-2 cells

**Figure 6:**
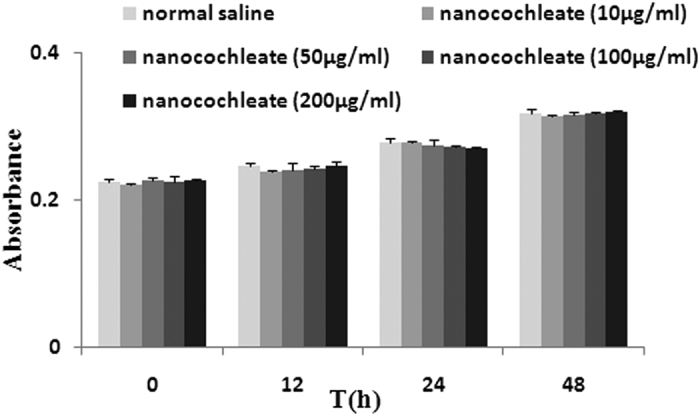
CCK-8 assay on Caco-2 cells (N = 3).

As indicated by the CCK-8assay, chitobiose-induced nanocochleates at a high concentration of 200 μg/mL for 48 h failed to induce any differences compared to the normal saline control group. Thus, Fig. 6 shows that Caco-2 cell proliferation was not significantly suppressed by chitobiose-induced nanocochleates.

**Figure 7:**
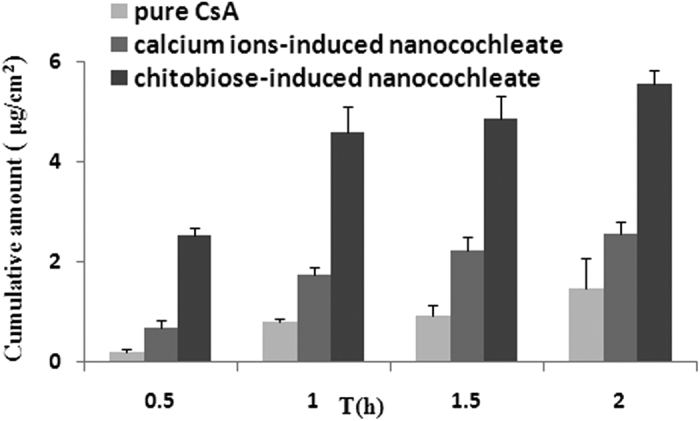
Transport profiles across the Caco-2 cell monolayer (N = 3).

As seen in Fig. 7, transfer efficiency of the three groups from the apical to basolateral side was in the following order: chitobiose-induced nanocochleates > calcium ion-induced nanocochleates > pure CsA. The transport profile of chitobiose-induced nanocochleates was increased 3.8- and 1.7-fold compared to pure CsA and calcium ion-induced nanocochleates, respectively. These data suggested that chitobiose-induced nanocochleates could significantly promote drug transport across the Caco-2 cell monolayer.

**Figure 8:**
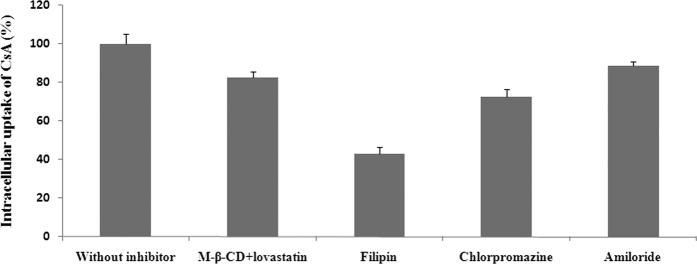
Transport profiles across Caco-2 cells after addition of different inhibitors (N = 3).

Figure 8 shows the transport profiles across Caco-2 cells after the addition of different inhibitors. Compared with the uptake in the control group, a 57.0% reduction of CsA in the presence of filipin indicated that Caco-2 uptake of chitobiose-induced nanocochleates mainly occurred through caveolae-mediated endocytosis. Chlorpromazine, as a clathrin inhibitor, is able to disrupt assembly and disassembly of clathrin, leading to reduced numbers of pit-associated receptors at the cell surface. A 17.2% reduction was also found in the transport of chitobiose-induced nanocochleates in the presence of chlorpromazine. CsA transport under conditions of cholesterol depletion was found to decrease slightly, suggesting that internalized pathways played a minor role in its uptake by Caco-2 cells. Finally, amiloride was used to study the potential effect of chitobiose-induced nanocochleates on Caco-2 cell macropinocytosis. Amiloridehad no significant effect on cellular uptake, implying that transport of chitobiose-induced nanocochleates across the apical membrane of Caco-2 cells did not occur via macropinocytosis.

**Figure 9:**
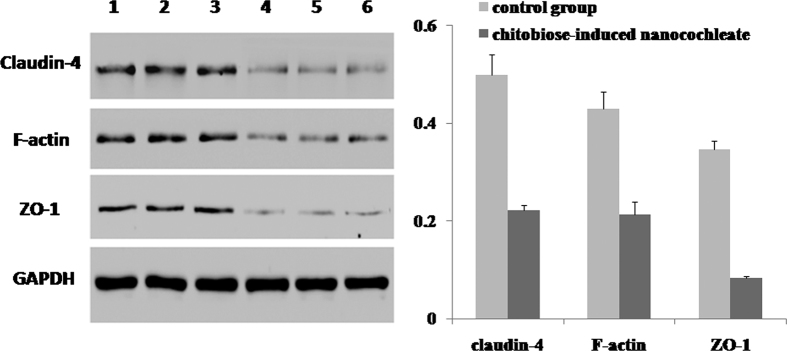
Western blot of ZO-1, F-actin and claudin-4 proteins in Caco-2 cells (N = 3). The PBS buffer solution group was the control group. 1–3: control group; 4–6: nanocochleate group.

After treatment with chitobiose-induced nanocochleates, three cellular tight junction proteins, including ZO-1, F-actin and claudin-4, were significantly down-regulated in Caco-2 cells (Fig. 9). This implied that chitobiose-induced nanocochleates could disrupt the integrity of cellular tight junctions in Caco-2 cells (Fig. S1), opening up a critical route for nanocochleate absorption from the gastrointestinal tract into the blood circulation.

## Discussion

It is important to find an effective approach to improve oral absorption of poorly water-soluble drugs. To our knowledge, this study is the first report that nanocochleate structures can assemble by the addition of chitobiosein the absence of calcium ions. Consequently, chitobiose has a dual role in nanocochleate formation and intestinal permeation. It represents a novel functional nanocochleate to overcome absorption barriers in the gastrointestinal tract.

As reported previously, nanocochleates are stable phospholipid-cation precipitates with multilayered cylindrical structures, composed of natural products, a divalent cation and negatively charged phospholipids^14^. The unique nanocochleate structure is characterized by spiral lipid sheets, in which a large, continuous, planar phospholipid bilayer sheet is rolled up in a spiral with little or no internal aqueous space. When calcium ions bind with phosphatidylserine head groups, nanoparticle internal water space is replaced, resulting in a restructuring of the lipid bilayer and formation of membrane discs that fuse into lipid-based supramolecular assemblies composed of rolled sheets of lipid bilayers and calcium. When drugs are incorporated into nanocochleates, the unique multilayered cylindrical structures provide protection from drug degradation in hazardous environmental conditions^15^.

The oligo-acyl-lysyl (OAK) as antimicrobial peptide could overcome multidrug resistance in bacteria by an efflux-enhanced resistance mechanism. An interesting study demonstrated thatthe multiple positively-charged OAK peptides induced nanocochleate formation of cylinders without the requirement for divalent cations^16^. Remarkably, OAK-induced nanocochleates increased drug therapeutic efficacy. In this case, OAK played a dual role: a passive role, driving nanocochleate formation, and an active role, enhancing bacterial sensitivity to encapsulated antibiotics against multidrug resistance by an efflux-enhanced resistance mechanism^17^. It is of interest to consider the importance of OAK-induced nanocochleates to give some of these systems unique properties in the presence of OAK^18^. Based on these findings, further options are available to optimize the pharmacological and biological characteristics of novel nanocochleate systems.

Chitosan, a polysaccharide derived from chitin, has been widely used in drug delivery systems because of it is non-toxic, has good biocompatibility and is biodegradable^19^,^20^. It can effectively increase drug oral absorption by contributing to tight adherence to and anchoring in the intestinal mucosal layer, prolonging gut residence time, penetrating into mucus and internalization by enterocytes, inhibiting the activity of tyrosine phosphatases, and reconstruction and opening of tightjunctions^21^. It has been reported that positively charged chitosan has remarkable capacity to cluster negatively charged glycoproteins through an electrostatic interaction^22^. Thus, we have developed a multifunctional nanocochleate to enhance oral absorption, in which a water-soluble chitosan derivative with a positive charge acts as a bridging agent of an anionic lipid bilayer by substituting for divalent cations.

It is of interest to consider what properties of chitosan with a positive charge are important to give some of these systems the unique property of promoting nanocochleate structures. One feature to consider is the charge on the lipid. Not only is this of interest because the negative charge on the lipid appears to play a key role in the formation of nanocochleates, but it has also been shown to influence dehydration of the lipid membrane interface and facilitate coiling of the lipid bilayer when bridged by cationic molecules^18^. Thus, five different water-soluble chitosanswith positive charge were selected to achieve a balance between charge and hydrophobicity. In this study, the addition of chitobiose, but not chitotetraose, low molecular weight chitosan, carboxymethyl chitosan, or chitosan oligosaccharide lactate, induced assembly of nanocochleates with the negatively charged DOPS. The typical multilayered cylindrical structure was confirmed by TEM images, indicative of chitobiose-induced nanocochleate formation in the absence of divalent cations.

In this work, the poorly water-soluble drug CsA was successfully incorporated into chitobiose-induced nanocochleates. The prepared chitobiose-induced nanocochleate containing CsA had a high entrapment efficiency of up to 81.4%. The introduction of chitobiose into the nanocochleates did not affect the particle size distribution, which was comparable to that of calcium ion-induced nanocochleates. However, there were significant differences in the *in vivo* and *in vitro* studies between the two formulations. The chitobiose-induced nanocochleate formulation of CsA exhibited a rapid release profile (up to 88.5% at 6 h) but release from the calcium ion-induced nanocochleate formulation was lower (68.9% at 6 h). The mechanism for the formation of calcium ion-induced nanocochleates assumes a fusion step mediated by Ca^2+^, in which the high efficiency of DOPS-Ca^2+^ interactions provides enough stability even when exposed to harsh environmental conditions. However, a slight difference may occur in chitosan-functionalized nanocochleates. Due to the relatively small positive charge on the chitobiose molecule, the DOPS-chitobiose interaction is weak compared to that of DOPS-Ca^2+^. One literature report suggested that several differently charged OAKs slowed formation of nanocochleates and affected their morphology and properties^17^. Consequently, chitobiose-induced nanocochleates were relatively easily dispersed in the medium, providing a large surface area. According to the Noyes-Whitney equation, the greater the surface area of the solute particles are, the higher the rate of dissolution. Therefore, the release rate from chitobiose-induced nanocochleates would be expected to be higher than from calcium ion-induced nanocochleates. Following CsA encapsulation by chitobiose-induced nanocochleates, its oral bioavailability was significantly higher compared to CsA solution and the calcium ion-induced nanocochleate formulation. Most effective absorption of chitobiose-induced nanocochleates was found in the duodenum and jejunum segments. Therefore, chitobiose-induced nanocochleate formulation constitutes a novel approach to enhance oral drug absorption.

Significant advances have led to our understanding of the potential absorption mechanisms of nanoparticles in the intestinal tract. The Caco-2 cell model, which mimics small intestine epithelium, is considered an effective tool for studying drug absorption^23^. Nanoparticle uptake by enterocytes is comprised of caveolae-mediated endocytosis, clathrin-mediated endocytosis, and clathrin- and caveolae-independent endocytosis^24^,^25^,^26^. In this study, transport of chitobiose-induced nanocochleates was markedly decreased after the addition of filipin or chlorpromazine. These data supported the conclusion that chitobiose-induced nanocochleates aretransported by Caco-2 cells mainly through caveolae-and clathrin-mediated endocytosis.

Tight junctions in intestinal epithelial cells consist of a largely impermeable barrier that hinders drug absorption from the gastrointestinal tract into blood. ZO-1^27^, F-actin^28^ and claudin-4^29^, three cellular tight junction proteins, were significantly down-regulated, indicating that chitobiose-induced nanocochleates could disrupt the integrity of cellular tight junctions in Caco-2 cells and open up a paracellular transport pathway to enhance intestinal absorption. The mechanism of this action can be explained by the following factors. Firstly, the positively charged chitosan rapidly adheres to the tight junction-associated protein on the cell membrane through an electrostatic interaction, followed by opening of the tight junctions^21^,^30^. Secondly, the positively charged chitosan can inhibit tyrosine phosphatases, leading to reconstruction and opening of the tight junctions^31^,^32^.

## Conclusion

In summary, we have successfully developed a novel nanocochleate formulation by the addition of chitobiose. Chitobiose-induced nanocochleates exhibit some promising properties to overcome intestinal barriers and improve oral absorption of CsA.

## Additional Information

**How to cite this article**: Liu, M. *et al*. Chitosan functionalized nanocochleates for enhanced oral absorption of cyclosporine A. *Sci. Rep.* **7**, 41322; doi: 10.1038/srep41322 (2017).

**Publisher's note:** Springer Nature remains neutral with regard to jurisdictional claims in published maps and institutional affiliations.

## Supplementary Material

Supplementary Figure S1

## Figures and Tables

**Figure 1:**
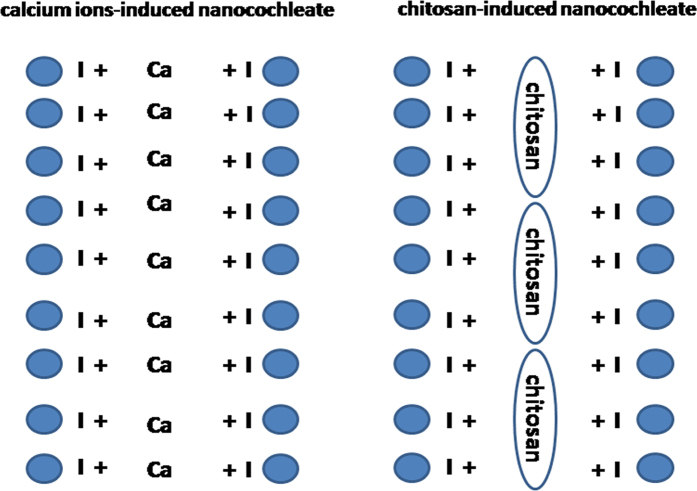
Schematic diagram of nanocochleates.

**Table 1:** Encapsulation efficiency of chitobiose-induced nanocochleates for CsA (N = 3).

lipid: drug (w/w)	Encapsulation efficiency (%)
50:1	71.8% ± 3.4%
10:1	81.4% ± 2.0%
5:1	60.8% ± 0.4%

**Table 2:** Particle size distribution of nanocochleates (N = 3).

Sample		mean particle size (nm)	PDI
Lipisomes		83.0 ± 1.6	0.07 ± 0.02
chitobiose-induced nanocochleate			
chitobiose	100 μl	106.7 ± 4.0	0.10 ± 0.01
chitobiose	500 μl	111.0 ± 2.0	0.08 ± 0.02
chitobiose	1000 μl	114.2 ± 0.8	0.09 ± 0.02
calcium ions-induced nanocochleate			
CaCl_2_	200 μl	104.0 ± 1.8	0.11 ± 0.06
CaCl_2_	400 μl	107.9 ± 0.6	0.08 ± 0.01
CaCl_2_	600 μl	113.5 ± 2.2	0.01 ± 0.02
