# Publisher Correction: Chitosan functionalized nanocochleates for enhanced oral absorption of cyclosporine A

**DOI:** 10.1038/s41598-019-41386-9

**Published:** 2019-04-03

**Authors:** Min Liu, Xiaoming Zhong, Zhiwen Yang

**Affiliations:** 1 Department of Pharmacy, Songjiang Hospital Affiliated Shanghai First People’s Hospital, Shanghai Jiao Tong University, Shanghai, China; 2 Urology Department, First Affiliated Hospital of Gannan Medical University, Gannan Medical University, Ganzhou, China; 3 Jiangxi Province Tumor Hospital, Nanchang, China

Correction to: *Scientific Reports* 10.1038/srep41322, published online 23 January 2017

This Article contains errors in the affiliation list, where Min Liu and Xiaoming Zhong are incorrectly affiliated with ‘Department of Pharmacy, Songjiang Hospital Affiliated Shanghai First People’s Hospital, Shanghai Jiao Tong University, Shanghai, China’.

The correct affiliation for Min Liu is listed below:

Urology Department, First Affiliated Hospital of Gannan Medical University, Gannan Medical University, Ganzhou, China

The correct affiliation for Xiaoming Zhong is listed below:

Jiangxi Province Tumor Hospital, Nanchang, China

