# Supplementary material for: Chitosan functionalized nanocochleates for enhanced oral absorption of cyclosporine A
Source: Sci Rep. 2017 Jan 23;7:41322. doi: 10.1038/srep41322 (PMC5282608; doi:10.1038/srep41322)
Supplement: Supplementary Figure S1 [file srep41322-s1.doc]

**Chitosan functionalized nanocochleate for enhanced oral absorption of cyclosporine A**

Min Liu ^b^*, Xiaoming Zhong^c^*, Zhiwen Yang^a^

a Department of Pharmacy, Songjiang Hospital Affiliated Shanghai First People’s Hospital, Shanghai Jiao Tong University, Shanghai, China

b **Urology Department, First Affiliated Hospital of Gannan Medical University, Gannan Medical University, Ganzhou,** China

c Jiangxi Province Tumor Hospital, Nanchang, China

Corresponding author: Zhiwen Yang.

*These authors contributed equally to this work

**Supplemental Figures**


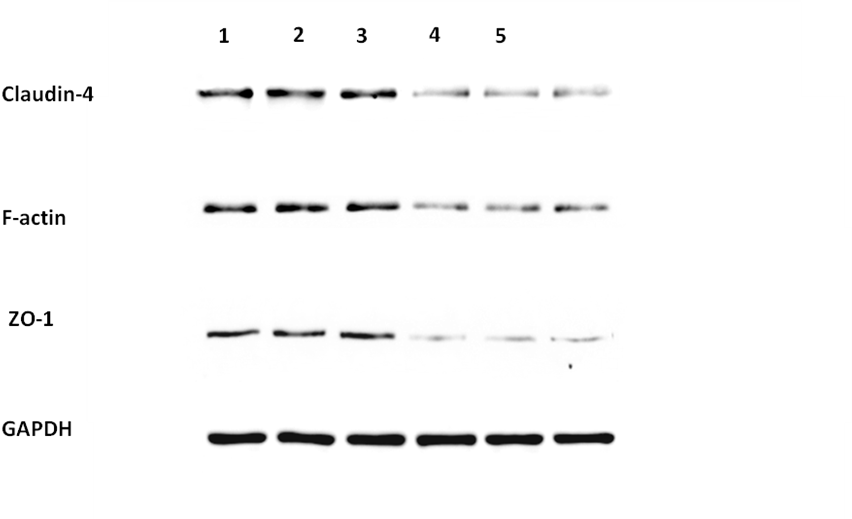


**Figure S1** . Western Blot of ZO-1、F-actin and claudin-4 proteins in Caco-2 cells.
